# Maternal anxiety forecasts shorter prolongation of pregnancies complicated by early-onset preeclampsia

**DOI:** 10.1007/s00404-022-06836-2

**Published:** 2022-11-25

**Authors:** Joris J. A. van Esch, Antoinette C. Bolte, Marc E. A. Spaanderman, Frank P. H. A. Vandenbussche, Carolina de Weerth, Roseriet Beijers

**Affiliations:** 1 Department of Obstetrics and Gynecology, Radboud University Medical Centre, Geert Grooteplein Zuid 10, Post 623, P.O. Box 9101, 6500 HB Nijmegen, The Netherlands; 2 Department of Cognitive Neuroscience, Donders Institute, Radboud University Medical Centre, Kapittelweg 29, 6525 EN Nijmegen, The Netherlands; 3 Department of Developmental Psychology, Behavioural Science Institute, Radboud University, P.O. Box 9104, 6500 HE Nijmegen, The Netherlands

**Keywords:** Early-onset preeclampsia, Stress, Anxiety, Hair cortisol, Pregnancy prolongation

## Abstract

**Purpose:**

In early-onset preeclampsia, each additional day of pregnancy prolongation reduces offspring infant mortality about 9%. We evaluated if maternal stress at admission to hospital for early-onset preeclampsia predicted admission-to-delivery intervals in days.

**Methods:**

This prospective, longitudinal cohort-study involved 15 singleton pregnancies with a diagnosis of preeclampsia before 34 weeks gestation with intended expectant management. Upon hospital admission, maternal psychological stress was assessed with questionnaires and physiological stress with hair cortisol. Hair samples were analyzed in three hair segments representing the preconception period, and the first and second trimester of pregnancy.

**Results:**

Mean pregnancy prolongation was 16.2 days. Higher maternal anxiety at hospital admission significantly correlated with shorter admission-to-delivery intervals (*r* = − 0.54, *p* = 0.04). Chronically increased hair cortisol concentrations (i.e. from preconception through the second trimester) of pregnancy tended to be related to shorter admission-to-delivery intervals (*p* <. 10).

**Conclusion:**

Higher reported anxiety is, and chronically high hair cortisol tended to be, related with fewer days of prolongation from admission to delivery in women with early-onset preeclampsia. These findings suggest that maternal stress might be a potential determinant of disease progression. Future research into early innovative stress-reducing interventions for early-onset preeclampsia may shed more light on the etiology and treatment of this disease.

## What does this study add to the clinical work

**Table:** 

The results of this first study with prospectively collected psychological and physiologic data relating maternal stress to pregnancy prolongation in women admitted to hospital for early-onset preeclampsia are innovative and of potential clinical value. Higher maternal anxiety at hospital admission predicted significantly shorter admission-to-delivery intervals.	

## Introduction

Preeclampsia affects 3% of all pregnancies [1, 2]. Preeclampsia occurring before 34 weeks of gestation (10% of preeclampsia cases) is considered early-onset and is associated with substantially increased maternal and neonatal morbidity and mortality [1, 3, 4]. The only known treatment for preeclampsia is delivery. Aggressive ‘interventionist management’ with delivery within 24–48 h, may benefit maternal outcome at the expense of increased neonatal morbidity and mortality. Conservative ‘expectant management’ to prolong pregnancy, may increase the risk for maternal complications, such as eclampsia, liver rupture, intracranial hemorrhage, pulmonary oedema, and kidney failure but may, however, improve offspring outcome [5, 6]. When interventionist management is compared to expectant management, the expectant approach results in decreased offspring morbidity; each additional day of prolongation reduces offspring infant mortality about 9% [7]. As such, expectant management is generally pursued in the Netherlands with continuous, vigilant surveillance of mother and fetus, with scheduled delivery for features of severe maternal disease or signs of fetal compromise [7, 8]. Little is known about maternal factors predicting shorter or longer prolongation. Knowing more about these factors may facilitate the development of future early interventions aimed at improving outcomes in women with early-onset preeclampsia. This study aims to investigate whether maternal psychological and physiological stress is associated with the number of days until delivery (i.e., admission-to-delivery intervals) in women hospitalized with early-onset preeclampsia.

In non-clinical populations, evidence is accumulating that maternal psychological stress and anxiety during pregnancy forecast adverse perinatal outcomes, including shorter gestations [9, 10]. A few studies investigated women hospitalized during pregnancy, and those most distressed were at highest obstetric risk [11, 12]. Next to measuring stress through self-report, stress can also be measured at a physiological level. When exposed to psychological or physical stress, the Hypothalamic–Pituitary–Adrenal (HPA)-axis is activated, resulting in the release of cortisol. Higher cortisol concentrations affect inflammatory responses, oxidative stress and endothelial function, and even uteroplacental blood flow, all correlates of gestational hypertensive disease [13–15]. These findings raise the question whether maternal psychological and physiological stress may be predictors of shorter or longer prolongation in early-onset preeclampsia.

The present study investigates maternal psychological and physiological stress upon hospital admittance with expectant management in women diagnosed with early-onset preeclampsia. Stress questionnaires included pregnancy-specific stress and anxiety, such as fear of bearing a child with disabilities, as these have also been related to adverse perinatal outcomes [9]. Physiological stress, i.e., HPA-axis activity, is assessed by cortisol concentrations in maternal hair. Hair cortisol permits evaluating cortisol output over longer periods of time [16, 17]. In this study, hair cortisol not only provides information about the concentrations during the disease (i.e. second trimester), but also allows preconception and first trimester concentrations to be determined. We hypothesized that higher psychological and physiological stress would be related to shorter admission-to-delivery intervals during expectant management in women hospitalized with early-onset preeclampsia.

## Methods

This study included women with early-onset preeclampsia (< 34 weeks gestation) hospitalized at a tertiary referral care center (Radboudumc, the Netherlands) during 2016–2017. The definition of the International Society for the Study of Hypertension in Pregnancy (ISSHP) [18] was used to define preeclampsia. Women with suspected preeclampsia were seen at our outpatient department. After confirmation of the diagnose of early-onset preeclampsia, women were admitted at the same day to the Obstetric High Care unit [4]. Corticosteroids for fetal lung maturation, antihypertensives and, magnesium sulphate, are administered according to the local protocol. At hospital admission, women with early-onset preeclampsia were asked to participate and after informed consent, filled in questionnaires and donated a sample of hair prior to corticosteroid treatment. Results of both assessments were blinded for participants and professionals. The study was approved by the local ethical committee, registered under CMO number 2015-2255 (NL-nr: 55946.091.15).

The primary outcome was prolongation of pregnancy in days from admission to delivery, representing the admission-to-delivery interval. According to national guidelines, in case of early-onset preeclampsia expectant management is advised until a gestational age of 37 weeks is reached. In case of severe deterioration of maternal or fetal conditions, it is at the discretion of the perinatal team or specialist on call to decide to induce the labor. Indication for delivery and prolongation of pregnancy were retrieved from the medical records. Independent variables were psychological stress and anxiety and hair cortisol concentrations. Validated questionnaires were used to measure general and pregnancy-specific stress and anxiety. General state anxiety was assessed by means of the State-Trait Anxiety Inventory (STAI) [19]. General daily hassles were assessed by means of a Dutch questionnaire: the Everyday Problem Checklist [20]. Pregnancy-specific anxiety was assessed by means of the Pregnancy-specific Anxieties Questionnaire–Revised (PRAQ-R) [21]. Two subscales were used, namely fear of giving birth, and fear of bearing a child with disabilities. Depressive symptoms were assessed by the Edinburgh Postnatal Depression Scale (EPDS) [22].

The hair sample was analyzed in 3-cm segments representing the cortisol concentrations in the preconception period (− 13–0 weeks), the first trimester (1–13 weeks) and the second trimester (14–26 weeks), as hair growths 1 cm per month [16, 17]. Corticosteroid therapy for fetal lung maturation did not affect hair cortisol, as the hair was cut before or immediately after corticosteroid administration. Cortisol concentrations were assayed at Dresden LAB Service GmbH, Germany, using a column-switching LC-APCI-MS/MS method [23]. Because of non-normality, log transformations were applied.

To investigate the associations between the admission-to-delivery intervals and the psychological stress and anxiety measures, (partial) correlations or regression analyses were performed, depending on the number of confounders that were (marginally) significantly related to prolongation. We considered the following confounders: pre-pregnancy body mass index, pregnancy smoking, maternal age, maternal educational level, gestational age upon admission, and objective maternal measures at admission (urine protein/creatinine ratio, systolic blood pressure, diastolic blood pressure, and HELLP syndrome).

To test whether prolongation of pregnancy was correlated to cortisol over the course of preconception to the second trimester, longitudinal regression analyses using mixed-model (multilevel) designs were performed. In these analyses, the hair cortisol measures representing the preconception period, first trimester, and second trimester were used at Level 1 and nested within the women at Level 2. Thereafter, a build-up strategy was followed in which variables (i.e. confounders, time, prolongation in days, and the interaction between time and prolongation) were added one-by-one to the model with random intercept and random slope. After adding each variable, the change in deviance on the -2log likelihood ratio scale after generalized least square estimations was assessed. Variables that did not improve the model by significantly reducing the deviance were excluded. The model with the best model fit is presented in the results.

## Results

### Descriptives

Baseline characteristics are reported in Table 1. We analyzed 15 cases of early-onset preeclampsia with a mean gestational age at admission of 30 weeks. The mean prolongation of pregnancy in days was 16.2 days, and in all cases, pregnancy was terminated at medical indication because of deterioration in maternal condition. Five women developed HELLP-syndrome, none eclampsia. All women received anti-hypertensive treatment, almost all with additionally magnesium sulphate.

**Table 1:** Sociodemographic and medical characteristics

	Mean Unless otherwise specified	SD	(Range)
*N*	15		
Maternal age	30.0	± 5.5	(19–40)
Parity			
Nulliparous (%)	87		
Parous^†^ (%)	13		
Ethnicity			
Caucasian (%)	93		
Body mass index			
Before pregnancy (kg/m^2^)	25	± 4.8	(20–35)
Maternal educational level			
Secondary education (%)	54		
College or university (%)	46		
Smoking in pregnancy			
Yes (%)	20		
Maternal descriptives at admission			
Preexistent hypertension^‡^ (%)	13		
Gestational age (weeks)	30.1	± 2.5	(24–33)
Systolic blood pressure (mmHg)	153	± 11	(175–140)
Diastolic blood pressure (mmHg)	100	± 8	(120–89)
Urine protein/creatinine ratio (g/10 mmol)	1.1	± 2.2	(0.30–8.10)
24-h urine protein (g/24 h)	4.3	± 4.3	(0.3–15.0)
HELLP-syndrome (%)	33.3		
Fetal grow restriction (%)	60.0		
Maternal descriptives during hospital stay			
Antihypertensive medications (*n*)	2		(1–3)
Intravenous magnesium sulfate (%)	87		
Highest urine protein/creatinine ratio after admission (g/10 mmol)	3.8	± 3.8	(0.3–11.9)
Eclampsia (%)	0		
Delivery outcomes			
Termination on maternal indication (%)	100		
Induction of labor, vaginal delivery (%)	40		
Induction of labor, assisted delivery (%)	7		
Caesarean section (%)	53		
Infant characteristics			
Gestational age at birth (weeks)	32.2	± 3.2	(25–37)
Birth weight (grams)	1612	± 518	(550–2848)
Small for gestational age (< p10)^§^ (%)	47		
NICU days	8	± 8.3	(0–29)
5’APGAR score	9	± 2.5	(0–10)
Primary outcome			
Prolongation of pregnancy (days)	16.2	± 10.5	(1–33)
Psychological stress questionnaires			
Anxiety (STAI)	42.9	± 5.7	(31–51)
Daily hassles (APL)	2.2	± 0.7	(0–3)
Fear of giving birth (PRAQ-R)	6.1	± 3.5	(3–15)
Fear of child with disabilities (PRAQ-R)	9.6	± 5.3	(4–20)
Depression (EPDS)	10.4	± 4.3	(4–16)
Cortisol in hair (nmol/l)			
Preconception	2.2	± 2.3	(0.4–9.0)
First trimester	3.3	± 3.0	(0.6–2.0)
Second trimester	7.2	± 5.9	(2.0–25.0)

*STAI* state-trait anxiety inventory (state was used), *APL* Dutch daily hassles questionnaire, *PRAQ-R* pregnancy-specific anxiety questionnaire–revised, *EPDS* Edinburgh Postnatal Depression Scale

^†^As one patient had an obstetric history of preeclampsia, we performed a sensitivity analysis excluding this patient from the main analyses; results remained similar; ^‡^As two patients had preexisting hypertension, we performed a sensitivity analysis excluding these two patients from the main analyses; results remained similar; ^§^Small for gestational age was defined as a birth weight below the 10th percentile of the new Dutch reference ‘Perined-Hoftiezer’ curves [24]

The mean score for general anxiety (STAI) was 42.93. Clinically significant anxiety scores, above 40 points [25], were reported in 12 out of 15 cases. The mean for depressive symptoms (EPDS) was 10.41. In 9 out of 15 patients, the score was ≥ 10, which indicates possible depression [22]. The remaining questionnaires do not have clinical cut-off values. Table 2 provides the correlations between the questionnaires, the hair cortisol measures, and pregnancy prolongation in days. Higher maternal anxiety at hospital admission predicted significantly less prolongation in days (*r* = − 0.54, *p* = 0.04), and higher hair cortisol concentrations during the second trimester tended to predict less prolongation in days (*r* = − 45, *p* = 0.05).

**Table 2:** Pearson correlations of predictor and outcome variables during pregnancy

	1	2	3	4	5	6	7	8	9
1. Days of pregnancy prolongation cortisol concentrations in hair	–								
2. Preconceptional period (− 13–0 wks)	− 0.35	–							
3. First trimester (0–13 wks)	− 0.37	0.87**	–						
4. Second trimester (13–26 wks) questionnaires of psychosocial stress	− 0.45*	0.65**	0.75**	–					
5. Anxiety (STAI)	− 0.54*	0.42	0.32	0.20	–				
6. Daily hassles (APL)	0.07	− 0.04	− 0.03	− 0.12	− 0.42	–			
7. Fear of giving birth (PRAQ-R)	0.03	0.10	− 0.01	− 0.18	0.28	0.06	–		
8. Fear of child with disabilities (PRAQ-R)	0.17	− .10	− 0.28	− 0.42	0.15	0.09	0.71*	–	
9. Depression (EPDS)	− 0.25	0.24	0.10	− 0.15	0.64*	0.16	0.41	0.32	–

*STAI* state-trait anxiety inventory, *APL* Dutch daily hassles questionnaire, *PRAQ-R* pregnancy-specific anxiety questionnaire–revised, *EPDS* Edinburgh Postnatal Depression Scale

*Correlation is significant at the 0.05 level (2-tailed); **Correlation is significant at the 0.01 level (2-tailed)

### Psychological stress and anxiety

**Fig. 1:**
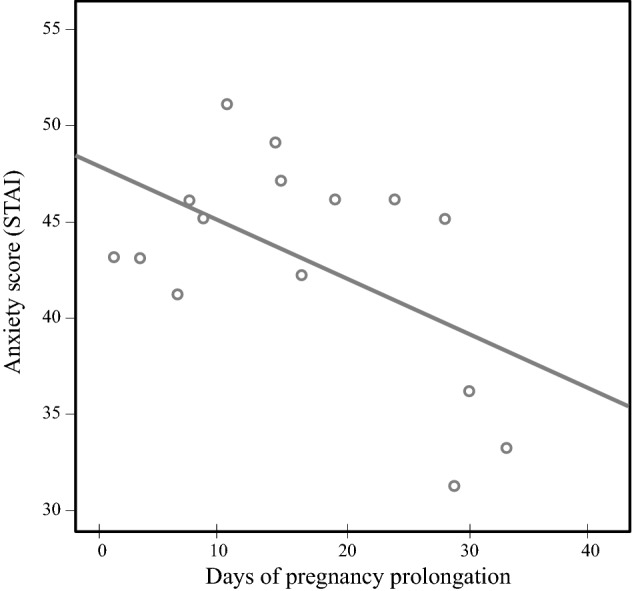
Scatterplot of correlation of anxiety and prolongation days (*r* = − 0.54, *p* = 0.04*)

As the confounders, including gestational age at hospital admittance, were not significantly related to admission-to-delivery interval (all *p* values > 0.378), we could rely on the correlations provided in Table 1. These correlations indicate that higher maternal anxiety, measured with the STAI at diagnosis, significantly forecasts fewer days of prolongation of pregnancy (Fig. 1). The other questionnaire measures were not significantly related to admission-to-delivery intervals.

### Cortisol in hair

**Fig. 2:**
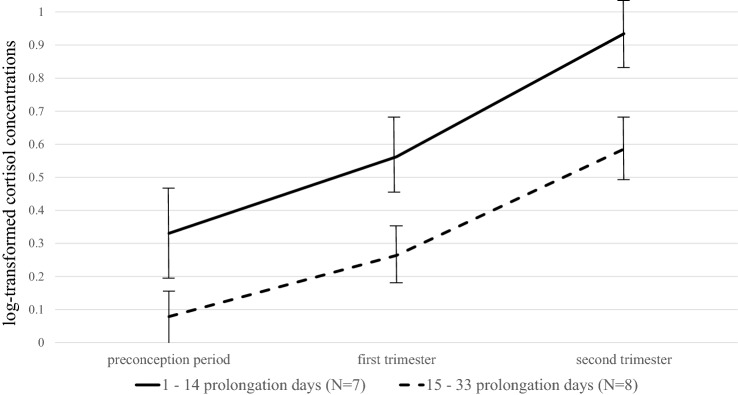
Visualization of the association between hair cortisol measurements over the course of pregnancy and prolongation days, divided in two groups

To investigate the associations between prolongation days and the hair cortisol measurements, multilevel analyses were performed. The final multilevel model is reported in Table 3. A significant effect of time was found, indicating that cortisol concentrations rise over the course of pregnancy, as was expected. A marginally significant negative effect of prolongation days on hair cortisol concentrations was found (*p* = 0.099). This effect of prolongation in days was not moderated by time, indicating that overall higher cortisol concentrations (from preconception till the second trimester), tended to be related to fewer prolongation days. To visualize these results, two groups were created (≤ 14 days prolongation, *N* = 7, versus ≥ 15 days prolongation, *N* = 8), and their mean hair cortisol concentrations were plotted (Fig. 2). This figure shows that the cortisol concentrations of the mothers with the fewest prolongation days was higher, from preconception till the second trimester.

**Table 3:** Estimates for the best fitting multilevel model for hair cortisol measurements

	Estimate	SE	*p* value
Intercept	0.10	0.15	0.492
Time	0.28	0.03	0.001**
Prolongation of pregnancy (days)	− 0.01	0.01	0.099

**Correlation is significant at the 0.01 level (2-tailed)

## Discussion

This study investigated whether maternal stress upon hospital admission was associated with fewer days of prolongation (i.e. admission-to-delivery intervals) in women hospitalized with early-onset preeclampsia. Stress was evaluated by stress and anxiety questionnaires, and by assessment of cortisol concentrations in hair. Higher anxiety, as reported by the participants at admission, correlated with shorter admission-to-delivery intervals. Moreover, higher overall hair cortisol, from preconception till the second trimester, was marginally related to shorter admission-to-delivery intervals. The other questionnaire measures were not significantly associated to prolongation days.

### Psychological stress and anxiety

The results are in accordance with previous studies in non-clinical populations showing that anxiety and stress during pregnancy are associated with shorter gestational duration [9, 15, 26]. Psychological stress could influence and increase systemic blood pressure by hyperstimulation of the sympathetic nervous system [15, 27]. Sympathetic overactivity has been observed in formerly preeclamptic women, with consequential sympathetically driven hyperdynamic circulation and increased shear stress on the vascular endothelium. These abnormal hemodynamic changes are thought to contribute to the hypertensive deterioration of pregnancy. Anti-hypertensive treatment may not be effective (enough) in these stressed, anxious patients, and preterm delivery might be unavoidable. Future research is needed to determine whether adding psychological stress-reducing interventions to the treatment of women with early-onset preeclampsia can extend admission-to-delivery intervals, as suggested in previous literature [28].

Whether the heightened anxiety at hospital admission was pre-existent or due to the situation is unknown. As prenatal stress and anxiety are stable in late pregnancy [19, 25], it is plausible that the women already suffered from anxiety. However, pregnant women facing acute medical crises or when admitted to hospital for pregnancy complications have been found to have higher levels of anxiety [12]. It is also possible that the feelings of anxiety are a result of the disease manifestation itself. The augmented sympathetic nervous system activity, seen in women with a history of preeclampsia [27], could be a pathophysiological explanation for the feelings of these women. The STAI-questionnaire statements ‘I am tense’ or ‘I feel strained’ for example, could describe these feelings and result in a high anxiety score. Therefore, stress-reducing interventions, such as cognitive behavioral therapy or mindfulness-based stress reduction therapy, may be effective in reducing symptoms and potentially positively affect the woman’s health. A randomized controlled trial in uncomplicated pregnancies with high reported Pregnancy-Related Anxiety indeed showed that mindfulness reduced maternal anxiety and improved pregnancy outcome [29]. These findings suggest that women prone to experience anxiety may profit from mindfulness-based stress reduction interventions, maybe even before pregnancy, to prevent pregnancy complications and negative outcomes.

### Cortisol in hair

In this study, hair cortisol concentrations were marginally associated to prolongation of pregnancy in early-onset preeclampsia. This effect was not moderated by time, indicating that overall higher cortisol concentrations, from preconception till the second trimester, tended to be related to shorter admission-to-delivery intervals. This is in line with a review showing that mothers delivering preterm had higher cortisol concentrations in blood or saliva at 27–37 weeks GA as compared to mothers delivering at term [10]. An increase in the release of cortisol and catecholamines triggers physiological responses, such as contractions of the peripheral blood vessels and an increase in blood pressure and heart rate, which in turn adversely affects the condition of mother and fetus. Cortisol concentrations in hair could possibly be indicating poor adaptation of the sympathetic nervous system to pregnancy in these women, resulting in a shorter admission-to-delivery interval in early-onset preeclampsia. Future research is needed to determine whether hair cortisol might be used as a biomarker of preconceptional risk estimation for pregnancy complications. Especially in women with a history of preeclampsia or a cardiovascular risk profile, as well as mental health complaints, it could be useful to know the risk of progression to severe disease in future pregnancies. Furthermore, preventive interventions are sorely needed for these women. Next to de-stressing psychological interventions mentioned earlier, aerobic training may be an effective intervention. Indeed, in a previous study on formerly pre-eclamptic women [27], aerobic training improved plasma volume expansion and reduced sympathetic overactivity, and as a result perhaps reduced a cause for stress.

### Associations between anxiety and cortisol during pregnancy

Both reported anxiety as well as hair cortisol concentrations (tended to be) related to shorter admission-to-delivery intervals. From the correlation table (Table 2), it can be seen that the reported anxiety measures were moderately related to hair cortisol concentrations (*r’s* = 0.20–0.42). This result is in line with previous studies who also found no or moderate correlations between maternal reports of stress/anxiety and cortisol measures during pregnancy [9, 30]. Note, however, that most previous studies measured cortisol in saliva, blood, and urine, reflecting more current and acute levels of cortisol, while the current study used hair cortisol. This enables retrospective examination of the cortisol production of the past months, representing more chronic levels of cortisol [17]. A study combining reported stress with hair cortisol measures in the first, second and third trimester in normotensive pregnancies [26], found a positive relation between self-reported stress (Perceived Stress Scale) at 16 weeks and second-trimester cortisol concentrations. Also, perceived stress at 16 weeks and second-trimester hair cortisol concentrations were positively associated with preterm delivery [26], suggesting that heightened cortisol might, to some extent, underlie links between psychological stress and pregnancy outcomes [9].

### Strengths and limitations

The strengths of this study are the prospectively collected psychological and physiologic data, and an innovative non-invasive biomarker of cortisol production covering the period from preconception to the second trimester. Also, we used multilevel analyses with repeated measures of hair cortisol to increase our statistical power. However, and in line with the rarity of early-onset preeclampsia, the size of the study is rather small and our results should be interpreted with caution. Future multi-center studies are needed to increase sample size and replicate our findings.

## Conclusions

The results of this first study relating maternal stress to pregnancy prolongation in women admitted to hospital for early-onset preeclampsia are innovative and of potential clinical value. The mean general anxiety scores of these women were above the cut-off for clinically significant anxiety. Moreover, higher maternal anxiety at hospital admission predicted significantly shorter admission-to-delivery intervals. Also, chronically increased hair cortisol concentrations (i.e. from preconception through the second trimester) were marginally significantly related to shorter admission-to-delivery intervals. Our findings regarding hair cortisol are contributing to a growing literature which suggests that chronic cortisol exposure may have important implications for health [17, 23, 26] and highlights the use of hair cortisol as a promising method for understanding the role of stress on pregnancy outcome. Future research into early innovative stress-reducing interventions for early-onset preeclampsia may shed more light on the etiology and treatment of this disease.

## Data Availability

Data of this population are requestable at the first author of this paper upon reasonable request.
